# Crystal structure of 6-chloro-5-iso­propyl­pyrimidine-2,4(1*H*,3*H*)-dione

**DOI:** 10.1107/S1600536814021382

**Published:** 2014-10-04

**Authors:** Nadia G. Haress, Hazem A. Ghabbour, Ali A. El-Emam, C. S. Chidan Kumar, Hoong-Kun Fun

**Affiliations:** a Department of Pharmaceutical Chemistry, College of Pharmacy, King Saud University, PO Box 2457, Riaydh 11451, Saudi Arabia; b King Abdullah Institute for Nanotechnology (KAIN), King Saud University, Riyadh 11451, Saudi Arabia; c X-ray Crystallography Unit, School of Physics, Universiti Sains Malaysia, 11800 USM, Penang, Malaysia

**Keywords:** crystal structure, pyrimidine-2,4-dione, hydrogen bonds, π–π inter­action

## Abstract

In the mol­ecule of the title compound, C_7_H_9_ClN_2_O_2_, the conformation is determined by intra­molecular C—H⋯O and C—H⋯Cl hydrogen bonds, which generate *S*(6) and *S*(5) ring motifs. The isopropyl group is almost perpendicular to the pyrimidine ring with torsion angles of −70.8 (3) and 56.0 (3)°. In the crystal, two inversion-related mol­ecules are linked *via* a pair of N—H⋯O hydrogen bonds into *R*_2_^2^(8) dimers; these dimers are connected into chains extending along the *bc* plane *via* an additional N—H⋯O hydrogen bond and weaker C—H⋯O hydrogen bonds. The crystal structure is further stabilized by a weak π–π inter­action [3.6465 (10) Å] between adjacent pyrimidine-dione rings arranged in a head-to-tail fashion, producing a three-dimensional network.

## Related literature   

For the biological activity of pyrimidine-2,4(1*H*,3*H*)-diones, see: Miyasaka *et al.* (1989 ▶); Tanaka *et al.* (1995 ▶); Hopkins *et al.* (1996 ▶); El-Brollosy *et al.* (2009 ▶); Klein *et al.* (2001 ▶); Nencka *et al.* (2006 ▶); El-Emam *et al.* (2004 ▶). For the use of 5-alkyl-6-chloro­pyrimidine-2,4(1*H*,3H)-diones in synthesis, see: El-Emam *et al.* (2004 ▶). For related pyrimidine-2,4-dione structures, see: El-Brollosy *et al.* (2011 ▶); Al-Omary *et al.* (2014 ▶); Haress *et al.* (2014 ▶). For the synthesis of the title compound, see: Al-Turkistani *et al.* (2011 ▶); Koroniak *et al.* (1993 ▶). For hydrogen-bond motifs, see: Bernstein *et al.* (1995 ▶).
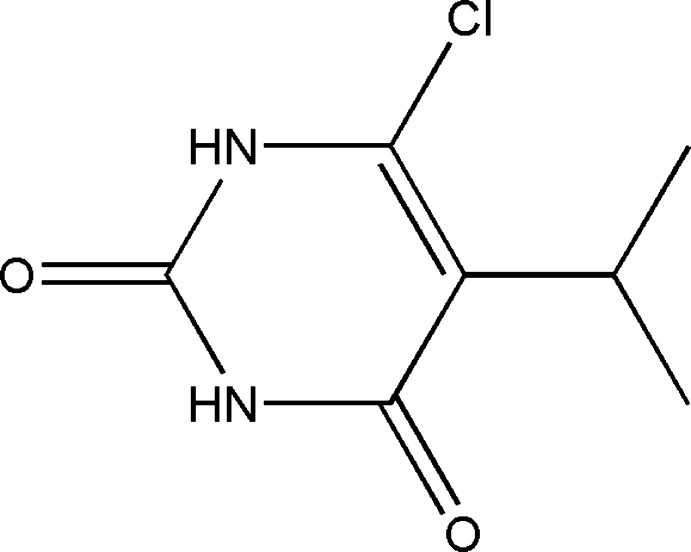


## Experimental   

### Crystal data  

C_7_H_9_ClN_2_O_2_
*M* *_r_* = 188.61
Monoclinic, 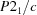

*a* = 11.2244 (4) Å
*b* = 6.8288 (3) Å
*c* = 11.6641 (5) Å
β = 104.577 (2)°
*V* = 865.26 (6) Å^3^
*Z* = 4
Cu *K*α radiation
μ = 3.62 mm^−1^
*T* = 296 K
0.45 × 0.28 × 0.26 mm

### Data collection  

Bruker APEXII CCD diffractometer
Absorption correction: multi-scan (*SADABS*; Bruker, 2009 ▶) *T*_min_ = 0.292, *T*_max_ = 0.458
5647 measured reflections
1553 independent reflections
1444 reflections with *I* > 2σ(*I*)
*R*_int_ = 0.027

### Refinement  

*R*[*F* ^2^ > 2σ(*F* ^2^)] = 0.040
*wR*(*F* ^2^) = 0.114
*S* = 1.06
1553 reflections
122 parameters
H atoms treated by a mixture of independent and constrained refinement
Δρ_max_ = 0.27 e Å^−3^
Δρ_min_ = −0.32 e Å^−3^

### 

Data collection: *APEX2* (Bruker, 2009 ▶); cell refinement: *SAINT* (Bruker, 2009 ▶); data reduction: *SAINT*; program(s) used to solve structure: *SHELXS97* (Sheldrick, 2008 ▶); program(s) used to refine structure: *SHELXL97* (Sheldrick, 2008 ▶); molecular graphics: *SHELXTL* (Sheldrick, 2008 ▶); software used to prepare material for publication: *SHELXTL* and *PLATON* (Spek, 2009 ▶).

## Supplementary Material

Crystal structure: contains datablock(s) global, I. DOI: 10.1107/S1600536814021382/sj5429sup1.cif

Structure factors: contains datablock(s) I. DOI: 10.1107/S1600536814021382/sj5429Isup2.hkl

Supporting information file. DOI: 10.1107/S1600536814021382/sj5429Isup3.cml

**Fig. 1.**
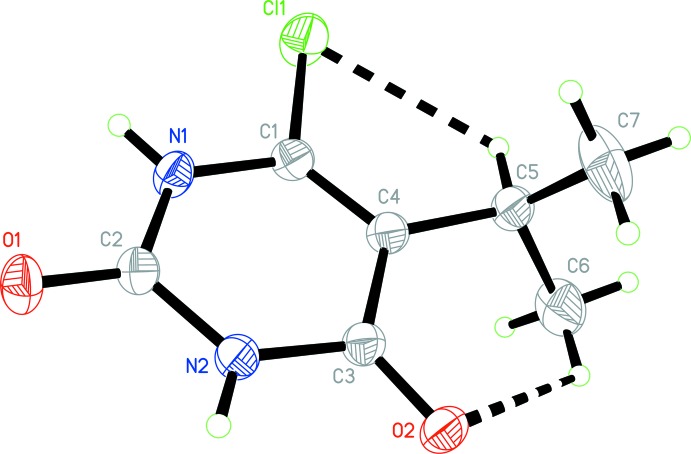
The molecular structure of the title compound with atom labels and 30% probability displacement ellipsoids.

. DOI: 10.1107/S1600536814021382/sj5429fig1.tif
The mol­ecular structure of the title compound with atom labels and 30% probability displacement ellipsoids.

**Fig. 2.**
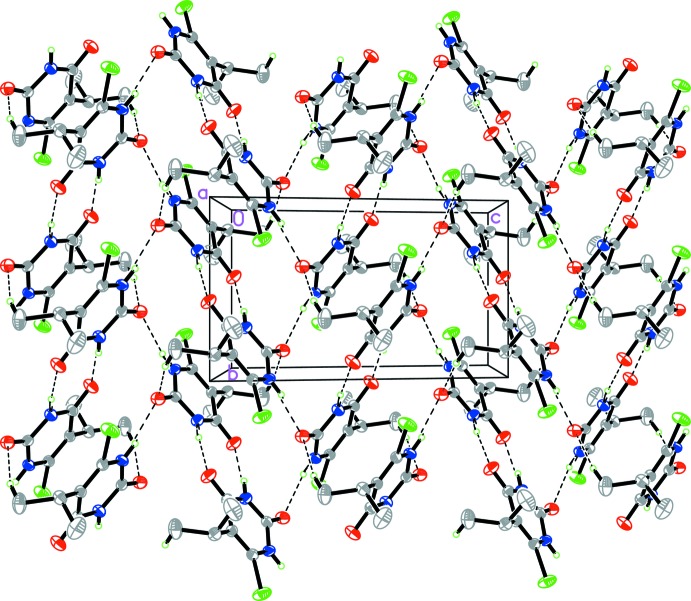
Crystal packing of the title compound, showing the hydrogen bonding interactions as dashed lines. H-atoms not involved in the hydrogen bonding are omited for clarity.

. DOI: 10.1107/S1600536814021382/sj5429fig2.tif
Crystal packing of the title compound, showing the hydrogen bonding inter­actions as dashed lines. H-atoms not involved in the hydrogen bonding are omited for clarity.

CCDC reference: 1026350

Additional supporting information:  crystallographic information; 3D view; checkCIF report

## Figures and Tables

**Table 1:** Hydrogen-bond geometry (, )

*D*H*A*	*D*H	H*A*	*D* *A*	*D*H*A*
N1H1*N*1O1^i^	0.83(3)	2.01(3)	2.833(2)	169(2)
N2H1*N*2O2^ii^	0.83(3)	2.03(3)	2.854(2)	171(2)
C5H5Cl1	0.94(3)	2.57(2)	3.132(2)	118.7(17)
C6H6*A*O1^iii^	0.96	2.56	3.455(3)	156
C6H6*B*O2	0.96	2.45	3.034(3)	119

Symmetry codes: (i) 
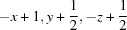
; (ii) 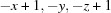; (iii) 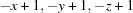.

## supplementary crystallographic information

### S1. Comment

Pyrimidine-2,4-diones and their related derivatives have long been known for their diverse chemotherapeutic activities including antiviral activity against HIV (Miyasaka *et al.*, 1989; Tanaka *et al.*, 1995; Hopkins *et al.*, 1996; El-Emam *et al.*, 2004). In addition, potent anticancer activity was observed for several pyrimidine-2,4-diones (Klein *et al.*, 2001; Nencka *et al.*, 2006). In a continuation of our interest in the chemical and pharmacological properties of pyrimidine and uracil derivatives (Al-Omary *et al.*, 2014; Haress *et al.*, 2014, El-Brollosy *et al.*, 2009), we have synthesized the title compound (I) as a precursor to the synthesis of a potential chemotherapeutic agent (Al-Turkistani *et al.*, 2011).

In the title compound (Fig. 1), the molecular conformation is stabilized by intramolecular C6–H6B···O2 and C5–H5···Cl1 hydrogen bonds incorporating *S*(6) and *S*(5) ring motifs respectively (Bernstein *et al.*, 1995). The isopropyl group is almost perpendicular to the N1/N2/C1–C4 ring with the C3–C4–C5–C7 and C3–C4–C5–C6 torsion angles of -70.8 (3)° and 56.0 (3)° respectively. In the crystal structure, two adjacent molecules are linked *via* a pair of N2–H1N2···O2 intermolecular hydrogen bonds forming inversion related *R*_2_^2^(8) dimers (Fig. 2).; these dimers are connected into chains *via* N1–H1N1···O1 and weak C6–H6A···O1 hydrogen bonds extending along the *bc* plane. The crystal structure is further stabilized by a weak π···π interaction [3.6465 (10) Å] producing a three-dimensional network.

### S2. Experimental

5-Isopropylbarbituric acid (8.51 g, 0.05 mol) was added portionwise with stirring to a mixture of phosphorus oxychloride (19.2 ml) and *N*,*N*-dimethyl aniline (10.3 ml) over a period of 10 minutes. The mixture was then heated under reflux for one hour. On cooling, the mixture was poured onto crushed ice (200 g m), stirred for 30 minutes and extracted with diethyl ether (400 ml). The ethereal extract was dried over anhydrous sodium sulfate and evaporated under vacuum at room temperature to yield the intermediate 5-isopropyl-2,4,6-trichloropyrimidine as a white waxy solid. 10% Sodium hydroxide (20 ml) was then added to the intermediate and the mixture was heated under reflux for 30 minutes. On cooling, the mixture was acidified with hydrochloric acid to pH 1–2 and the separated precipitate was filtered, washed with cold water and crystallized from ethanol to yield 6.98 g (74%) of the title compound (C_7_H_9_ClN_2_O_2_) as colourless crystals. M.P.: 257–259 °C.

^1^H NMR (DMSO-d_6_, 500.13 MHz): δ 1.14 (d, 6H, CH_3_, J = 7.2 Hz), 2.51–2.63 (m, 1H, CH), 11.22 (s, 1H, NH), 11.79 (s, 1H, NH). ^13^C NMR (DMSO-d_6_, 125.76 MHz): δ 20.02 (CH_3_), 26.52 (CH), 113.95 (C–5), 140.95 (C-6), 149.75 (C=O), 162.75 (C=O).

### S3. Refinement

The nitrogen-bound H-atoms were located in a difference Fourier map and were refined freely [N–H 0.83 (2) and 0.84 (3) Å]. Other H atoms were positioned geometrically (C=H 0.95–0.96 Å) and refined using a riding model with *U*_iso_(H) = 1.2 *U*_eq_(C) or 1.5 *U*_eq_(C) for methyl H atoms. A rotating group model was used for the methyl groups.

### Crystal data

**Table:**

C_7_H_9_ClN_2_O_2_	*F*(000) = 392
*M**_r_* = 188.61	*D*_x_ = 1.448 Mg m^−^^3^
Monoclinic, *P*2_1_/*c*	Cu *K*α radiation, λ = 1.54178 Å
Hall symbol: -P 2ybc	Cell parameters from 3341 reflections
*a* = 11.2244 (4) Å	θ = 3.9–69.4°
*b* = 6.8288 (3) Å	µ = 3.62 mm^−^^1^
*c* = 11.6641 (5) Å	*T* = 296 K
β = 104.577 (2)°	Block, colourless
*V* = 865.26 (6) Å^3^	0.45 × 0.28 × 0.26 mm
*Z* = 4	

### Data collection

**Table:**

Bruker APEXII CCD diffractometer	1553 independent reflections
Radiation source: fine-focus sealed tube	1444 reflections with *I* > 2σ(*I*)
Graphite monochromator	*R*_int_ = 0.027
φ and ω scans	θ_max_ = 69.7°, θ_min_ = 4.1°
Absorption correction: multi-scan (*SADABS*; Bruker, 2009)	*h* = −12→13
*T*_min_ = 0.292, *T*_max_ = 0.458	*k* = −8→7
5647 measured reflections	*l* = −13→10

### Refinement

**Table:**

Refinement on *F*^2^	Secondary atom site location: difference Fourier map
Least-squares matrix: full	Hydrogen site location: inferred from neighbouring sites
*R*[*F*^2^ > 2σ(*F*^2^)] = 0.040	H atoms treated by a mixture of independent and constrained refinement
*wR*(*F*^2^) = 0.114	*w* = 1/[σ^2^(*F*_o_^2^) + (0.0664*P*)^2^ + 0.3263*P*] where *P* = (*F*_o_^2^ + 2*F*_c_^2^)/3
*S* = 1.06	(Δ/σ)_max_ < 0.001
1553 reflections	Δρ_max_ = 0.27 e Å^−^^3^
122 parameters	Δρ_min_ = −0.32 e Å^−^^3^
0 restraints	Extinction correction: *SHELXL*, Fc^*^=kFc[1+0.001xFc^2^λ^3^/sin(2θ)]^-1/4^
Primary atom site location: structure-invariant direct methods	Extinction coefficient: 0.0181 (15)

### Special details

**Table:**

Geometry. All e.s.d.'s (except the e.s.d. in the dihedral angle between two l.s. planes) are estimated using the full covariance matrix. The cell e.s.d.'s are taken into account individually in the estimation of e.s.d.'s in distances, angles and torsion angles; correlations between e.s.d.'s in cell parameters are only used when they are defined by crystal symmetry. An approximate (isotropic) treatment of cell e.s.d.'s is used for estimating e.s.d.'s involving l.s. planes.
Refinement. Refinement of *F*^2^ against ALL reflections. The weighted *R*-factor *wR* and goodness of fit *S* are based on *F*^2^, conventional *R*-factors *R* are based on *F*, with *F* set to zero for negative *F*^2^. The threshold expression of *F*^2^ > σ(*F*^2^) is used only for calculating *R*-factors(gt) *etc*. and is not relevant to the choice of reflections for refinement. *R*-factors based on *F*^2^ are statistically about twice as large as those based on *F*, and *R*- factors based on ALL data will be even larger.

### Fractional atomic coordinates and isotropic or equivalent isotropic displacement parameters (Å^2^)

**Table:**

	*x*	*y*	*z*	*U*_iso_*/*U*_eq_	
Cl1	0.20279 (5)	0.71985 (9)	0.34179 (6)	0.0644 (3)	
O1	0.55744 (12)	0.3674 (2)	0.28760 (12)	0.0465 (4)	
O2	0.35879 (13)	0.0719 (2)	0.53323 (13)	0.0522 (4)	
N1	0.39383 (14)	0.5227 (2)	0.32786 (14)	0.0386 (4)	
N2	0.45727 (14)	0.2261 (2)	0.41291 (14)	0.0394 (4)	
C1	0.29636 (16)	0.5181 (3)	0.37878 (16)	0.0371 (4)	
C2	0.47498 (15)	0.3710 (3)	0.33892 (15)	0.0354 (4)	
C3	0.36295 (16)	0.2134 (3)	0.46972 (15)	0.0371 (4)	
C4	0.27390 (15)	0.3729 (3)	0.44806 (15)	0.0368 (4)	
C5	0.16037 (17)	0.3635 (3)	0.49593 (17)	0.0437 (5)	
C6	0.1871 (2)	0.3338 (5)	0.6280 (2)	0.0697 (7)	
H6A	0.2438	0.4322	0.6675	0.105*	
H6B	0.2226	0.2065	0.6480	0.105*	
H6C	0.1119	0.3437	0.6527	0.105*	
C7	0.0719 (2)	0.2127 (5)	0.4302 (3)	0.0815 (9)	
H7A	0.0568	0.2355	0.3465	0.122*	
H7B	−0.0041	0.2217	0.4533	0.122*	
H7C	0.1065	0.0845	0.4486	0.122*	
H1N1	0.403 (2)	0.617 (4)	0.2856 (19)	0.043 (6)*	
H1N2	0.506 (2)	0.132 (4)	0.422 (2)	0.059 (7)*	
H5	0.125 (2)	0.489 (4)	0.481 (2)	0.058 (7)*	

### Atomic displacement parameters (Å^2^)

**Table:**

	*U*^11^	*U*^22^	*U*^33^	*U*^12^	*U*^13^	*U*^23^
Cl1	0.0662 (4)	0.0486 (4)	0.0892 (5)	0.0249 (2)	0.0395 (3)	0.0243 (3)
O1	0.0481 (7)	0.0421 (8)	0.0587 (9)	0.0001 (6)	0.0307 (6)	−0.0012 (6)
O2	0.0522 (8)	0.0506 (9)	0.0614 (9)	0.0157 (6)	0.0286 (6)	0.0227 (7)
N1	0.0458 (8)	0.0321 (8)	0.0424 (9)	0.0018 (6)	0.0197 (7)	0.0042 (6)
N2	0.0394 (8)	0.0381 (8)	0.0451 (9)	0.0095 (7)	0.0187 (7)	0.0061 (6)
C1	0.0392 (9)	0.0358 (9)	0.0379 (10)	0.0053 (7)	0.0124 (7)	−0.0011 (7)
C2	0.0373 (8)	0.0343 (9)	0.0365 (9)	−0.0012 (7)	0.0129 (7)	−0.0044 (7)
C3	0.0376 (9)	0.0394 (10)	0.0361 (10)	0.0033 (7)	0.0125 (7)	0.0025 (7)
C4	0.0380 (9)	0.0393 (10)	0.0346 (9)	0.0044 (7)	0.0120 (7)	0.0005 (7)
C5	0.0420 (9)	0.0460 (11)	0.0485 (11)	0.0095 (8)	0.0215 (8)	0.0060 (8)
C6	0.0585 (13)	0.108 (2)	0.0512 (14)	0.0016 (14)	0.0296 (11)	−0.0019 (13)
C7	0.0472 (13)	0.120 (3)	0.0849 (19)	−0.0208 (14)	0.0314 (13)	−0.0335 (17)

### Geometric parameters (Å, º)

**Table:**

Cl1—C1	1.7200 (18)	C4—C5	1.516 (2)
O1—C2	1.222 (2)	C5—C7	1.500 (3)
O2—C3	1.226 (2)	C5—C6	1.507 (3)
N1—C2	1.364 (2)	C5—H5	0.95 (3)
N1—C1	1.370 (2)	C6—H6A	0.9600
N1—H1N1	0.83 (2)	C6—H6B	0.9600
N2—C2	1.360 (2)	C6—H6C	0.9600
N2—C3	1.386 (2)	C7—H7A	0.9600
N2—H1N2	0.84 (3)	C7—H7B	0.9600
C1—C4	1.342 (3)	C7—H7C	0.9600
C3—C4	1.457 (2)		
			
C2—N1—C1	121.92 (16)	C7—C5—C6	111.5 (2)
C2—N1—H1N1	117.6 (15)	C7—C5—C4	110.49 (17)
C1—N1—H1N1	120.4 (15)	C6—C5—C4	114.38 (17)
C2—N2—C3	126.87 (16)	C7—C5—H5	109.6 (16)
C2—N2—H1N2	116.1 (17)	C6—C5—H5	106.3 (15)
C3—N2—H1N2	116.9 (17)	C4—C5—H5	104.3 (15)
C4—C1—N1	124.69 (16)	C5—C6—H6A	109.5
C4—C1—Cl1	123.17 (14)	C5—C6—H6B	109.5
N1—C1—Cl1	112.13 (13)	H6A—C6—H6B	109.5
O1—C2—N2	123.07 (17)	C5—C6—H6C	109.5
O1—C2—N1	122.60 (17)	H6A—C6—H6C	109.5
N2—C2—N1	114.32 (15)	H6B—C6—H6C	109.5
O2—C3—N2	119.22 (16)	C5—C7—H7A	109.5
O2—C3—C4	124.43 (16)	C5—C7—H7B	109.5
N2—C3—C4	116.35 (16)	H7A—C7—H7B	109.5
C1—C4—C3	115.60 (16)	C5—C7—H7C	109.5
C1—C4—C5	123.76 (16)	H7A—C7—H7C	109.5
C3—C4—C5	120.54 (16)	H7B—C7—H7C	109.5
			
C2—N1—C1—C4	3.4 (3)	N1—C1—C4—C5	−175.22 (17)
C2—N1—C1—Cl1	−175.53 (14)	Cl1—C1—C4—C5	3.6 (3)
C3—N2—C2—O1	−176.48 (18)	O2—C3—C4—C1	177.94 (19)
C3—N2—C2—N1	4.4 (3)	N2—C3—C4—C1	−2.5 (2)
C1—N1—C2—O1	175.02 (17)	O2—C3—C4—C5	−5.7 (3)
C1—N1—C2—N2	−5.8 (2)	N2—C3—C4—C5	173.89 (16)
C2—N2—C3—O2	179.34 (18)	C1—C4—C5—C7	105.3 (2)
C2—N2—C3—C4	−0.3 (3)	C3—C4—C5—C7	−70.8 (3)
N1—C1—C4—C3	1.0 (3)	C1—C4—C5—C6	−128.0 (2)
Cl1—C1—C4—C3	179.85 (13)	C3—C4—C5—C6	56.0 (3)

### Hydrogen-bond geometry (Å, º)

**Table:**

*D*—H···*A*	*D*—H	H···*A*	*D*···*A*	*D*—H···*A*
N1—H1*N*1···O1^i^	0.83 (3)	2.01 (3)	2.833 (2)	169 (2)
N2—H1*N*2···O2^ii^	0.83 (3)	2.03 (3)	2.854 (2)	171 (2)
C5—H5···Cl1	0.94 (3)	2.57 (2)	3.132 (2)	118.7 (17)
C6—H6*A*···O1^iii^	0.96	2.56	3.455 (3)	156
C6—H6*B*···O2	0.96	2.45	3.034 (3)	119

Symmetry codes: (i) −*x*+1, *y*+1/2, −*z*+1/2; (ii) −*x*+1, −*y*, −*z*+1; (iii) −*x*+1, −*y*+1, −*z*+1.
