# Supplementary figures and images for: Crystal structure of 6-chloro-5-iso­propyl­pyrimidine-2,4(1H,3H)-dione
Source: Acta Crystallogr Sect E Struct Rep Online. 2014 Oct 4;70(Pt 11):o1144–5. doi: 10.1107/S1600536814021382 (PMC4257309; doi:10.1107/S1600536814021382)

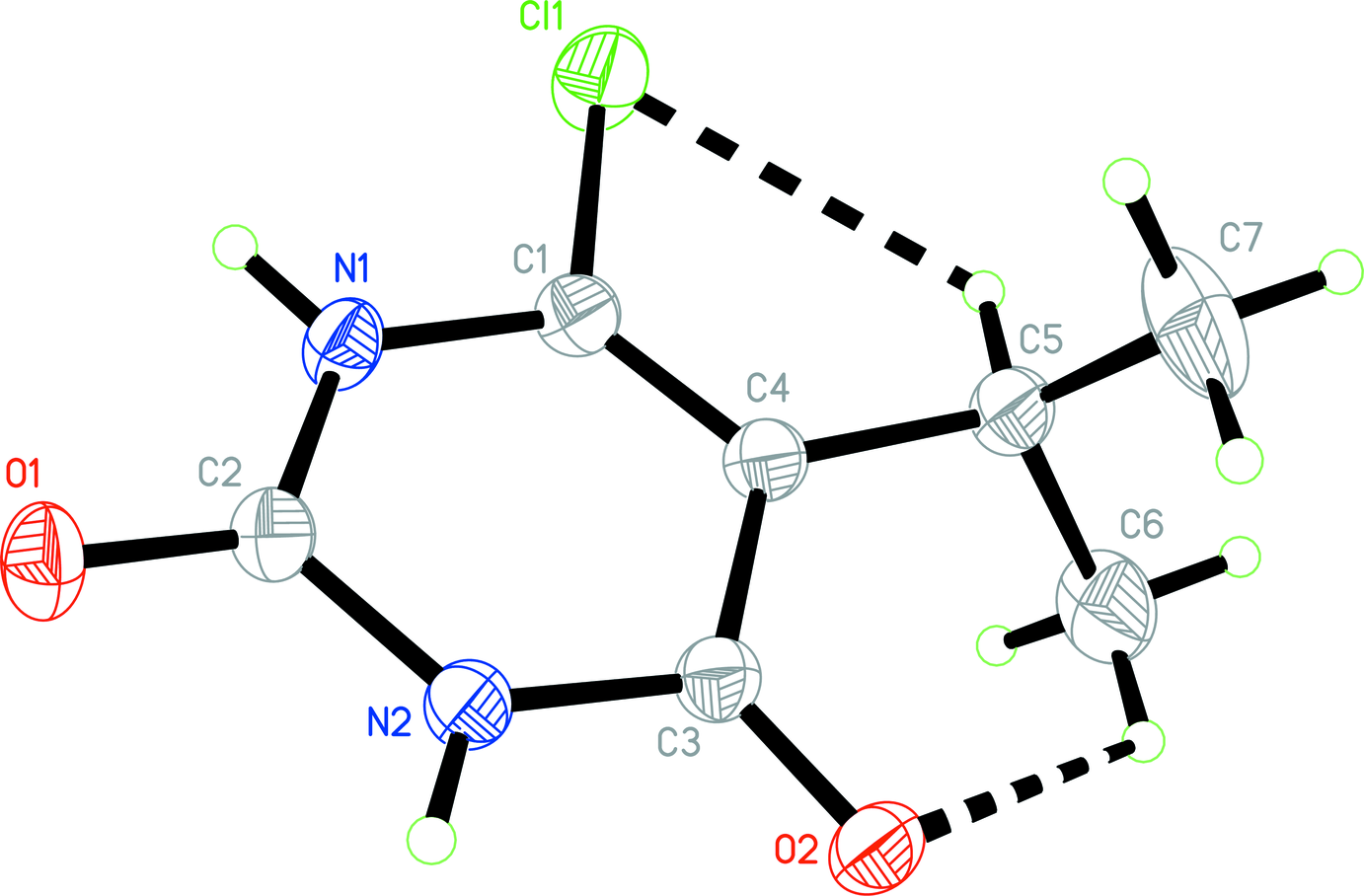

Supplement: Supplementary file 4 [file e-70-o1144-fig1.tif]

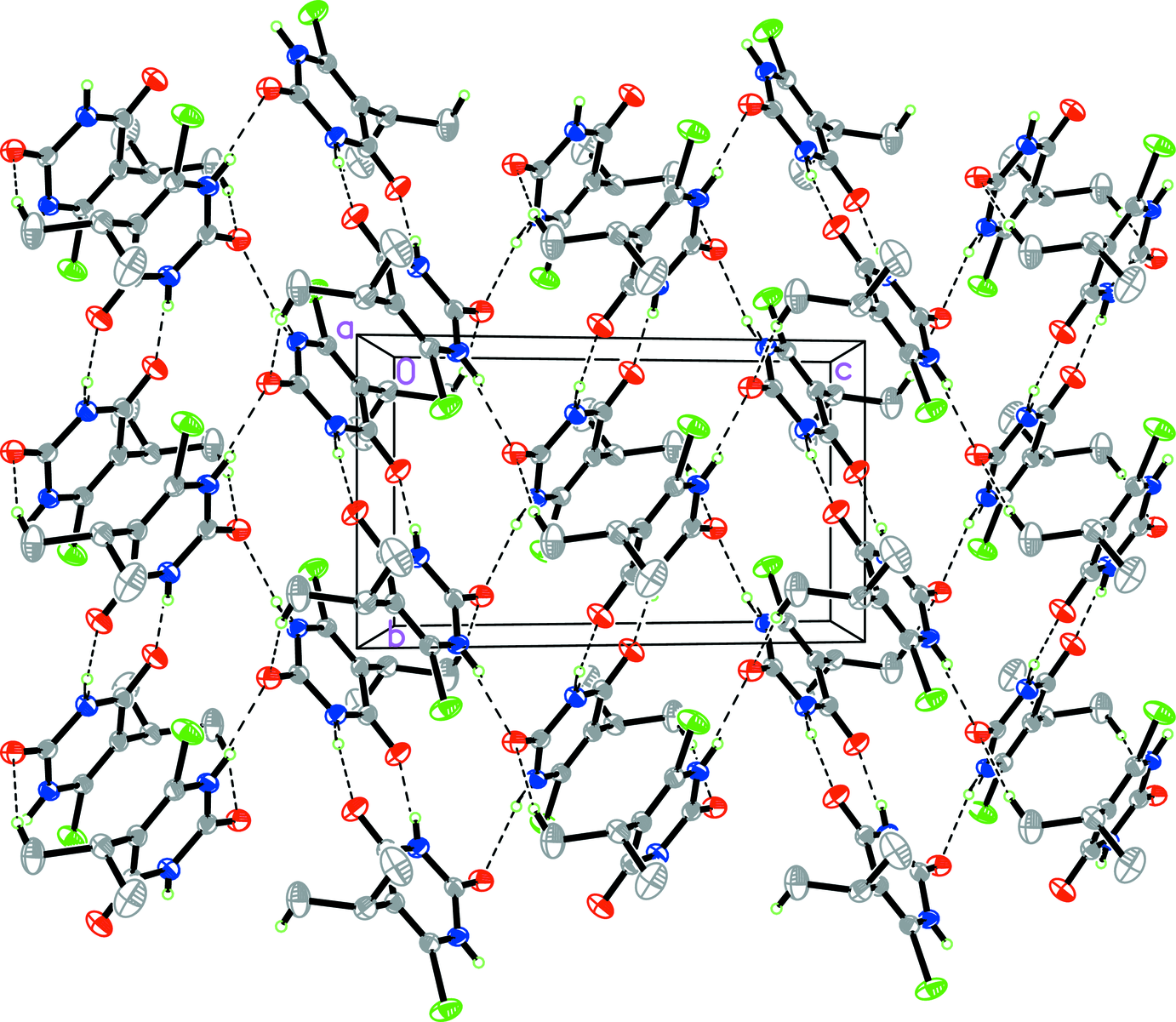

Supplement: Supplementary file 5 [file e-70-o1144-fig2.tif]
